# Molecular Basis of the Extracellular Ligands Mediated Signaling by the Calcium Sensing Receptor

**DOI:** 10.3389/fphys.2016.00441

**Published:** 2016-09-30

**Authors:** Chen Zhang, Cassandra L. Miller, Rakshya Gorkhali, Juan Zou, Kenneth Huang, Edward M. Brown, Jenny J. Yang

**Affiliations:** ^1^Department of Chemistry, Georgia State UniversityAtlanta, GA, USA; ^2^Center for Diagnostics and Therapeutics, Georgia State UniversityAtlanta, GA, USA; ^3^Division of Endocrinology, Diabetes and Hypertension, Department of Medicine, Brigham and Women's HospitalBoston, MA, USA

**Keywords:** calcium sensing receptor, cooperativity, amino acids, structure, trafficking, disease mutations

## Abstract

Ca^2+^-sensing receptors (CaSRs) play a central role in regulating extracellular calcium concentration ([Ca^2+^]_o_) homeostasis and many (patho)physiological processes in multiple organs. This regulation is orchestrated by a cooperative response to extracellular stimuli such as small changes in Ca^2+^, Mg^2+^, amino acids, and other ligands. In addition, CaSR is a pleiotropic receptor regulating several intracellular signaling pathways, including calcium mobilization and intracellular calcium oscillation. Nearly 200 mutations and polymorphisms have been found in CaSR in relation to a variety of human disorders associated with abnormal Ca^2+^ homeostasis. In this review, we summarize efforts directed at identifying binding sites for calcium and amino acids. Both homotropic cooperativity among multiple calcium binding sites and heterotropic cooperativity between calcium and amino acid were revealed using computational modeling, predictions, and site-directed mutagenesis coupled with functional assays. The hinge region of the bilobed Venus flytrap (VFT) domain of CaSR plays a pivotal role in coordinating multiple extracellular stimuli, leading to cooperative responses from the receptor. We further highlight the extensive number of disease-associated mutations that have also been shown to affect CaSR's cooperative action via several types of mechanisms. These results provide insights into the molecular bases of the structure and functional cooperativity of this receptor and other members of family C of the G protein-coupled receptors (cGPCRs) in health and disease states, and may assist in the prospective development of novel receptor-based therapeutics.

## Introduction

**Figure 1:**
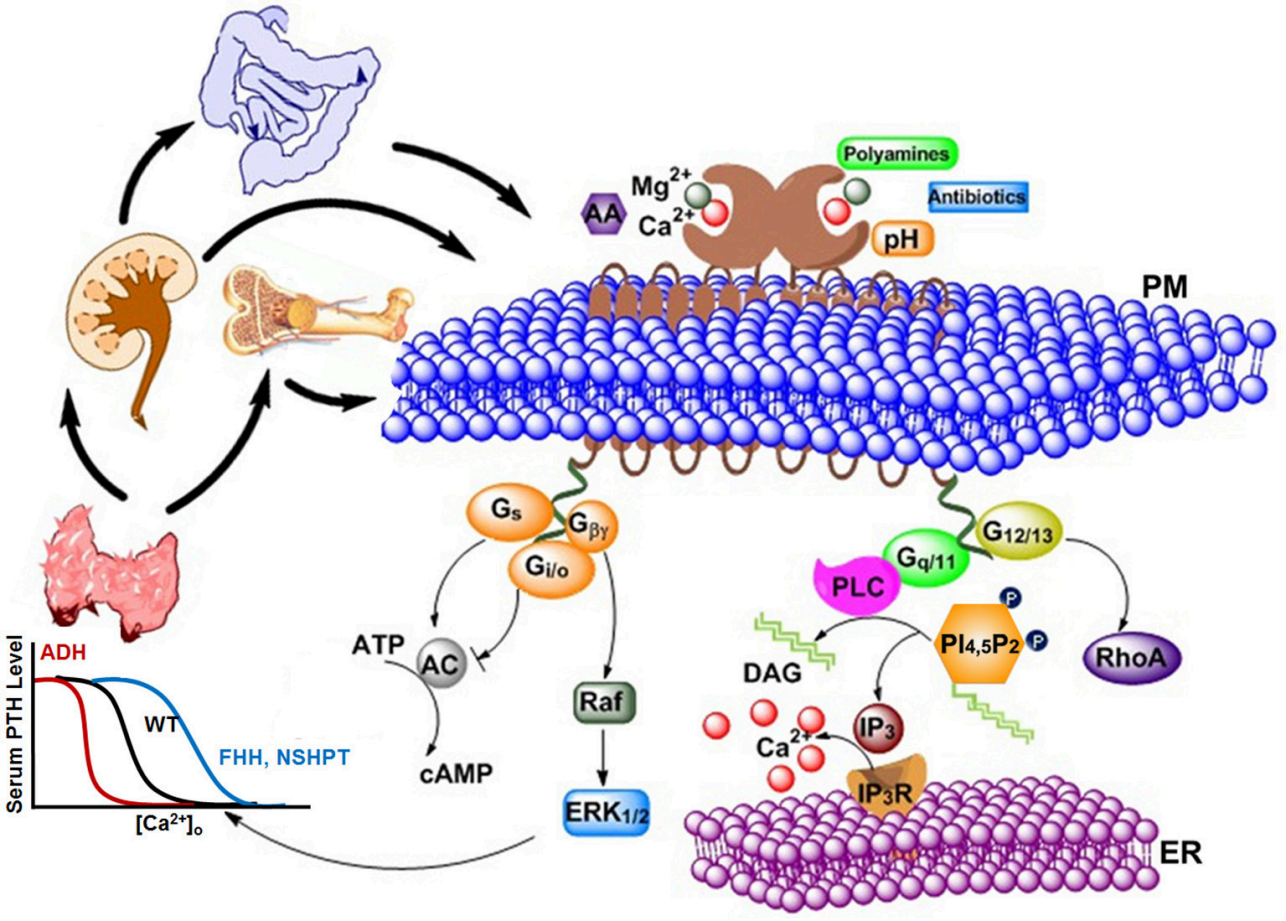
**Various types of agonists, including cations, peptides, amino acids, antibiotics, etc., can act on the extracellular calcium-sensing receptor (CaSR) to generate a complex intracellular signaling network**. CaSR is also a pleiotropic receptor in its regulation of four G protein-mediated intracellular signaling pathways (G_q∕11_, G_i∕o_, G_s_, and G_12∕13_). The correlations and crosstalk among different signaling cascades contribute the cooperative responses of intracellular calcium responses as well as parathyroid hormone (PTH) secretion (left) and intracellular calcium responses (right) to extracellular calcium. Bottom left: The sigmoidal relationship between calcium concentration in blood and PTH level in serum is demonstrated. Higher Ca^2+^ concentration is required for normal level of PTH in patients with familial hypocalciuric hypercalcemia (FHH) or patients with Neonatal Severe Primary Hyperparathyroidism (NSHPT), as the response curve shifts to the right; on the other hand, lower Ca^2+^ concentration than normal is enough to trigger PTH secretion in patients with autosomal dominant hypocalcemia (ADH). PM, Plasma membrane; ER, Endoplasmic reticulum; AA, arachidonic acid; AC, adenylate cyclase; cAMP, cyclic AMP; DAG, diacylglycerol; ERK_1∕2_, extracellular-signal-regulated kinase; G_s_, G_i∕o,_G_12∕13,_ and G_q∕11_, subunits of the s-, i-, 12/13, and q-type alpha subunit of heterotrimeric G proteins, respectively; G_βγ_, G beta and gamma complex; IP3, inositol-1,4,5-trisphosphate; IP_3_R, inositol-1,4,5-trisphosphate receptor; PLC, phospholipase C; PI(4,5)P_2_, phoshatidylinositol-4,5-bisphosphate; RhoA, Ras homolog gene family, member A.

In 1883, Sydney Ringer serendipitously discovered Ca^2+^ to be essential for the contraction of isolated hearts (Ringer, 1883). After the mid-twentieth century, research on Ca^2+^ has grown at an exponential rate, and Ca^2+^ is now considered as a universal signal carrier for biological information (Krebs and Michalak, 2007). Ca^2+^ controls life and death, as it modulates the process of fertilization as well as apoptosis. Ca^2+^ was primarily considered as a crucial secondary messenger via rapidly and efficiently regulated changes in intracellular calcium levels, and modulated extensive molecular signaling components through calcium channels, exchangers along with pumps. The discovery of the calcium sensing receptor (CaSR) defined an additional role of extracellular Ca^2+^ as a first messenger (Smajilovic and Tfelt-Hansen, 2007). It is known that serum Ca^2+^ concentration can regulate the secretion of parathyroid hormone (PTH) and, therefore, research was pursued to investigate how this process is accomplished. In 1993, Dr. Edward M. Brown cloned CaSR, which is primarily responsible for this type of regulation, from bovine parathyroid gland (Brown et al., 1993). The previously observed cytosolic Ca^2+^ changes in parathyroid cells, as well as in other *in vitro* expression systems, were triggered by changes in serum Ca^2+^ concentration and have been largely proven to be mediated by the CaSR (Nemeth and Scarpa, 1987; Muff et al., 1988). In addition to the key tissues involved in extracellular Ca^2+^ and Mg^2+^ homeostasis (e.g., parathyroid, thyroid, kidney, bone), CaSR has also been reported to be present in diverse other, non-homeostatic tissues (e.g., brain, skin, etc.) (Lundgren et al., 1994; Hebert, 1996; Cima et al., 1997; Hinson et al., 1997; Cheng et al., 1998; Kovacs et al., 1998; Chattopadhyay et al., 1999; Brown and MacLeod, 2001; Buchan et al., 2001; Mathias et al., 2001; Hofer and Brown, 2003; Chang and Shoback, 2004; Fudge and Kovacs, 2004; Hofer et al., 2004). To date, extracellular Ca^2+^ has been shown to be a first messenger via CaSR and 14 other family cGPCRs, including metabotropic glutamate receptors (mGluRs) and γ-aminobutyric acid (GABA)_B_ receptors. As shown in Figure 1, high [Ca^2+^]_o_ triggers multiple CaSR-regulated intracellular signaling pathways, including G_q∕11_ signaling, G_i∕o_signaling, G_s_ signaling, extracellular signal-regulated kinases 1 and 2 (ERK_1∕2_) signaling, and intracellular calcium mobilization.

CaSR is comprised of 1078 amino acids, and is encoded by exons 2–7 to encompass a 19 amino acids (AA) N-terminal signal peptide, a ~600 AA extracellular domain (ECD), a cysteine rich domain (CRD), a ~250 AA 7-transmembrane (7TM) domain, and the ~216 AA intracellular domain (ICD) (Pollak et al., 1993; Chikatsu et al., 2000; Hendy et al., 2013). The ECD of CaSR not only plays crucial roles in the sensing of nutrients, such as Ca^2+^, L-Phe and polypeptides, allowing ligands to modulate CaSR cooperatively, but is also essential for the dimerization of the receptor (Ray et al., 1999; Zhang et al., 2014b). Binding of Ca^2+^ and other CaSR ligands to the ECD is thought to produce conformational changes in the 7TM domain, introducing alterations in intracellular loops and the C-terminal domain, which further trigger the downstream signaling pathways (Brown and MacLeod, 2001). The intracellular C-terminal domain (residues 863–1078) is quite diverse among species. It participates in controlling the CaSR signaling and its cooperativity, modulating receptor trafficking, expression and desensitization (Gama and Breitwieser, 1998; Bai, 2004; Ward, 2004; Huang et al., 2006).

A unique characteristic of CaSR is the high cooperativity of Ca^2+^ dependent activation, which tightly controls the secretion of parathyroid hormone when the receptor is exposed to serum Ca^2+^ concentrations within its responsive range and also regulates the delicate Ca^2+^ homeostasis within the body as a whole (Brown et al., 1993). Functional cooperativity of CaSR (i.e., based on biological activity determined using functional assays), particularly the functional positive homotropic cooperative response to extracellular calcium, is essential for the receptor's ability to respond over a narrow physiological range of [Ca^2+^]_o_ (1.1–1.3 mM) (Breitwieser, 2006). CaSR has an estimated Hill coefficient of 3–4 for its regulation of biological processes, such as activating intracellular Ca^2+^ signaling, inhibiting PTH release in parathyroid cells (Figure 1) and stimulating calcitonin secretion in C-cells (Walter et al., 2013). Other extracellular mineral cations, such as Mg^2+^, and amino acids, are able to function as agonists and co-agonists to regulate/potentiate the Ca^2+^-induced activation of the CaSR. L-amino acids, especially aromatics, at physiological conditions potentiate the Ca^2+^–elicited activation of the CaSR by altering the EC_50_ values for extracellular calcium evoked intracellular calcium responses via positive heterotropic functional cooperativity (Conigrave et al., 2000; Francesconi and Duvoisin, 2004; Wang et al., 2006). This capacity of CaSRs to integrate both divalent cations and other extracellular stimuli, such as amino acids (Vetter and Lohse, 2002), is a feature shared by other cGPCRs (Wise et al., 1999; Galvez et al., 2000; Gether, 2000; Oldham and Hamm, 2008; Rosenbaum et al., 2009). In this paper, we will review the major discoveries and approaches that have been taken in uncovering the molecular basis of the cooperative responses of CaSR and its impact in understanding molecular bases of diseases (Zhang et al., 2014b). Key determinants contributing to the functional cooperativity of CaSR including the calcium and ligand binding sites, and the connectivity between distant sites on the ECD domain as well as protein expression at the membrane surface and intracellular domain will be discussed.

## Challenges

To date, how Ca^2+^, Mg^2+^, and amino acids cooperatively modulate intracellular Ca^2+^ signaling is a long-standing unanswered question mainly due to the lack of a determined CaSR structure. Like other cGPCRs, CaSR functions as a dimer (Bai et al., 1998a, 1999; Pace et al., 1999; Kunishima et al., 2000; Zhang et al., 2001; Bai, 2004; Suzuki et al., 2004) with a very long N-terminus that is predicted to fold into a bilobed ECD (Brown et al., 1993; Hebert and Brown, 1995; Pearce et al., 1996; Hinson et al., 1997; Hofer and Brown, 2003; Hu and Spiegel, 2003; Jingami et al., 2003; Quinn et al., 2004). The ECD has been shown to play an important role in the cooperative responses of the receptor to changes of [Ca^2+^]_o,_ amino acids, metabolites, and neurotransmitters (Bai, 1999, 2004; Conigrave et al., 2002; Zhang et al., 2002; Chang and Shoback, 2004; Conigrave and Lok, 2004; Mun et al., 2004). Determination of the X-ray structure of the ECD of CaSR is largely hampered by difficulty in crystallization due to heterogeneous and extensive glycosylation (11 potential N-glycosylation sites) and challenges associated with membrane proteins (Yang et al., 2005). Further, Ca^2+^ and ligand-binding sites with weak binding affinities and rapid off rates are often not occupied in a determined X-ray structure. For example, no bound Ca^2+^ has been observed in over 30 X-ray structures of the ECD of mGluRs (Kunishima et al., 2000; Tsuchiya et al., 2002; Jiang et al., 2010; Zhang et al., 2014a), despite the clear modulatory effect of [Ca^2+^]_o_ on this receptor. Furthermore, additional challenges result from the lack of direct binding assays for weak Ca^2+^-binding, amino acid-binding (K_d_ ~mM) and limitations in obtaining purified membrane proteins with native conformations (Nagar et al., 1996; Hu and Spiegel, 2003; Magno et al., 2011). The quantification of functional cooperativity with binding cooperativity and visualization of the molecular connectivity in tuning cooperativity requires innovative approaches.

## Modeling structures of the CaSR

Several regions of the CaSR and mGluRs are highly homologous, providing the basis for molecular modeling of the structure of CaSR under various conditions based on the determined crystal structures of mGluRs with and without ligands or agonists/antagonists (Bai, 2004; Hu and Spiegel, 2007; Huang Y. et al., 2007). Various mGluR structures have indicated that agonist binding induces a rearrangement of the dimeric VFT by bringing the two monomers closer together owing to pulling the lower lobes inwards upon the change toward the closed state. This is further evidenced by the addition of gadolinium, which is found to likely stabilize the active form of mGluR1 (Tsuchiya et al., 2002). Recent crystal structures of various mGluRs have been solved that shed light on several possible sequential activation events. Orthosteric ligands bind to the VFT in the dimer, stabilizing the closed conformation to induce an activation signal to the transmembrane domain by the CRD, which then activates G protein-regulated signaling pathways (Kunishima et al., 2000; Jingami et al., 2003; Muto et al., 2007; Wu et al., 2014).

**Figure 2:**
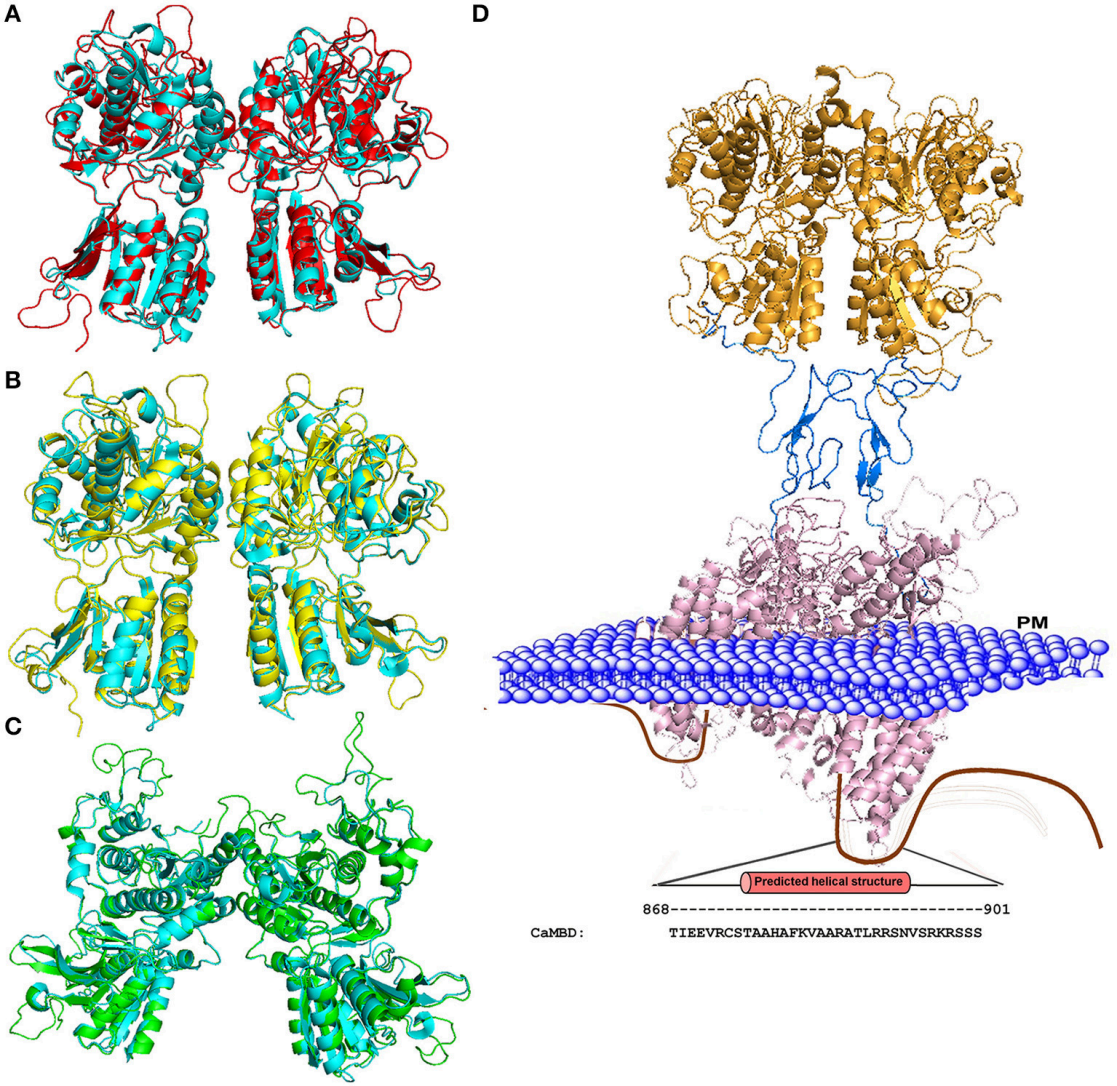
**Homology models of the CaSR ECD. (A)** Structural alignment of the mGluR1 holo form (cyan; PDB ID 1EWK, mGluR1 with glutamate) with the modeled structure of CaSR (red). **(B)** Structural alignment of mGluR1 apo form I (cyan, PDB ID 1EWV) with the modeled structure of CaSR (yellow). **(C)** Structural alignment of mGluR1 apo form II (cyan, PDB ID 1EWT) with the modeled structure of CaSR (green). **(D)** Homology model of the full CaSR structure. The ECD is based on the crystal structure of CaSR, the cysteine rich domain modeled from mGluR3 (PDB ID 2E4U), and the 7TM domain is modeled from mGluR1 with a ligand (PDB ID 4OR2). The C-terminal of CaSR can interact with protein kinases, ubiquitin ligase, CaM, etc. The region including residues from 868 to 901 is predicted to be CaM binding site. Mutations on the CaM binding site compromise the stability of surface expressed CaSR (Huang et al., 2010). PM: Plasma Membrane.

Figure 2 shows three modeled structures of the ECD of CaSR in different conformational states based on the crystal structure of mGluRs. The ECD of CaSR possesses a featured bilobed VFT structure similar to mGluRs and bacterial periplasmic binding proteins (O'Hara et al., 1993; Ray et al., 1999; Reyes-Cruz et al., 2001). These modeled structures of CaSR derived from several crystal structures of mGluR1 provide molecular views of large conformational variances among different states and therefore provide a potential functional mechanism of CaSR and by extension, of other cGPCRs. The VFT region alternates between closed and open states, determined by the presence of a ligand (e.g., glutamate for mGluR1 or Ca^2+^ for CaSR), which then causes the signal to be relayed to the 7TM domain. As cGPCRs are only functional as dimers, the dimerization of CaSR is likely to be driven by the hydrophobic interactions between the monomers along the dimer interface. CaSR and mGluR protomers are also covalently linked by several disulfide bridges between the two monomers. A ligand induced rearrangement of the dimeric VFT brings the two monomers closer together by pulling the lower lobes inwards upon the change toward the closed state. Similar to mGluR, the ligand was proposed to interact with both lobes of the VFT to stabilize the closed form by providing additional points of contact between the lobes (Kunishima et al., 2000).

The homology models of the CaSR 7TM domain was built based on the crystal structure of bovine rhodopsin by several independent research groups (Petrel et al., 2003; Miedlich et al., 2004; Hu et al., 2006; Bu et al., 2008). Miedlich et al. docked the CaSR with the antagonist, NPS 2143, and the calcimimetic, NPS R-568, to the derived 7TM domain model structure and reported a shared pocket composed of residues F668, F684 and E837 (Miedlich et al., 2004). The evidence that there are additional Ca^2+^-binding sites located at the 7TM domain of the CaSR (7TM) have been provided by different independent groups (Hauache et al., 2000; Hu et al., 2002, 2005). The ECD portion of CaSR, including the CRD linker, has a 30–35% sequence similarity across all mGluRs, while the linker alone has 65–70% similarity (Bai, 2004). Together with a recent determination of structure of the ECD domain of mGluR3 with the cysteine-rich domain and the transmembrane domain of mGluR1 makes it possible to model the full length CaSR shown in Figures 2C,D. Due to high sequence homology, the modeled transmembrane structure provides more structural information than previous modeled 7TM domains based on the crystal structure of bovine rhodopsin (Petrel et al., 2003; Miedlich et al., 2004; Hu et al., 2006; Bu et al., 2008). These modeling and structural studies, therefore, provide important insights into the large conformational changes induced by agonist binding to both mGluR and CaSR. For instance, the ECD is changed from an open status to a closed form, although the calcium binding sites and amino acid binding sites remain elusive due to their weak binding affinities.

## Identification of Ca^2+^ binding sites in the ECD and their homotropic cooperativity

**Figure 3:**
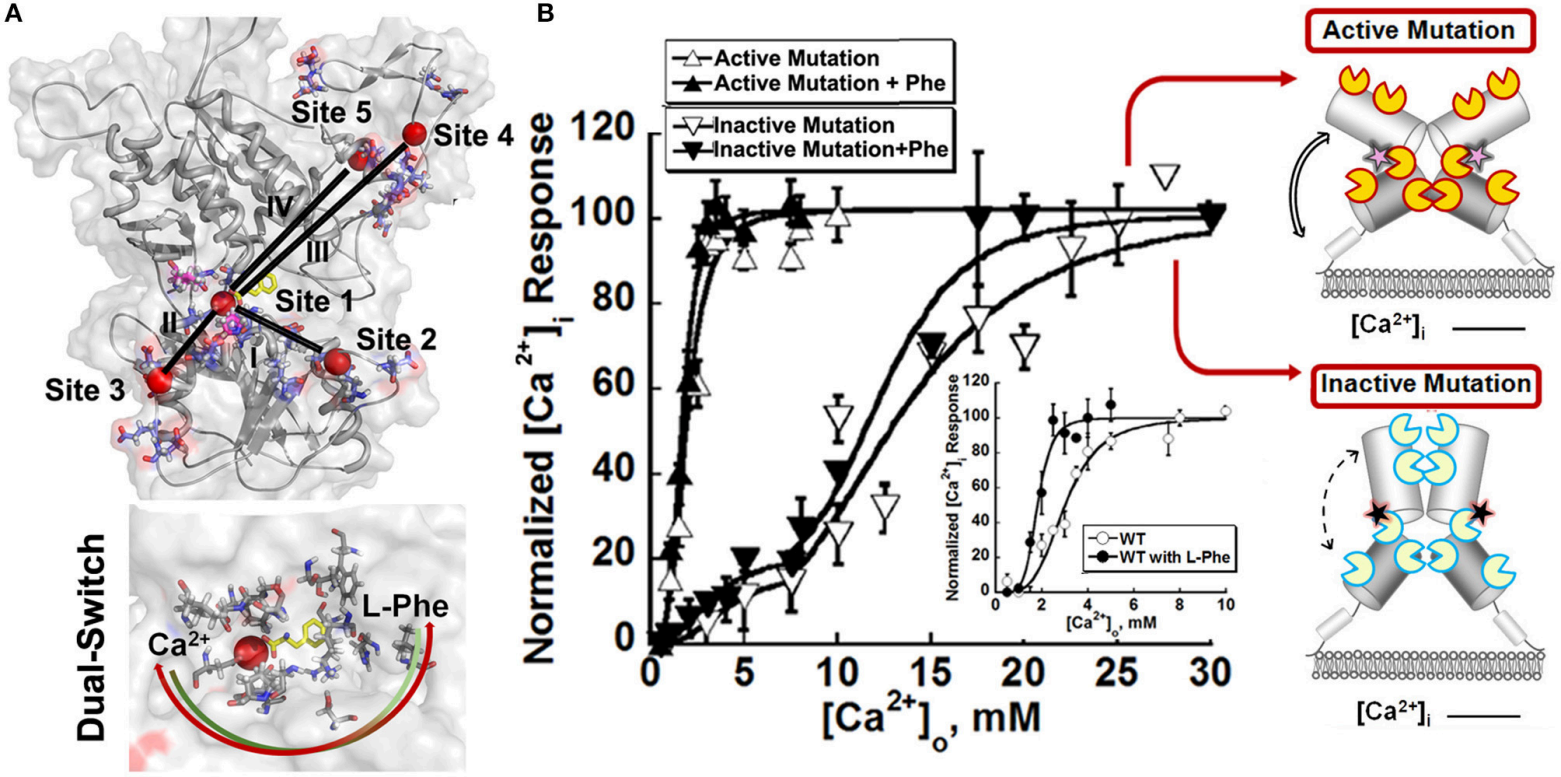
**Molecular connectivity and heterotrophic cooperativity. (A)** The correlated motions between calcium binding site 1 and the other calcium binding sites, designated by roman numbers (I~V), are mapped onto the CaSR ECD models. The correlation map and parameters for molecular dynamics simulation can be found in the paper (Zhang et al., 2014a). Bottom: The hetero-communication between Ca^2+^ and an amino acid functions as a dual switch that enhances the function of CaSR by positively impacting multiple Ca^2+^-binding sites within the ECD. Red sphere: Ca^2+^. Two headed arrow: both ligands can affect the binding of the other, therefore, the downstream signaling pathways. **(B)** The intracellular Ca^2+^ responses of HEK293 cells transiently overexpressing an active mutation or an inactive mutation. Inset: Representative response of WT CaSR to the changes of extracellular Ca^2+^ in the absence or presence of L-Phe, showing homotropic, and heterotropic cooperativity. Some disease mutations can interrupt the homotropic cooperativity shown as intracellular calcium responses to changes in the extracellular calcium concentration as well as the heterotropic cooperativity in the presence of allosteric modulators (e.g., L-Phe). Open circles: in the absence of L-Phe; Closed circles: in the presence of 5 mM L-Phe. Yellow “pacman”: Ca^2+^ binding sites in an active receptor; Blue “pacman”: Ca^2+^ binding sites in an inactive receptor. Pink stars: gain-of-function mutations in the hinge region. Black stars: loss-of-function mutations in the hinge region. Two headed arrows with solid lines: enhanced correlation motions between different Ca^2+^ binding sites. Two headed arrows with dash lines: impaired correlation motions.

Since the ECD is responsible for the main binding activity, researchers turned to identifying the binding regions for Ca^2+^ and amino acids based on studies from homology modeling, mutational studies and the effect of disease related mutations (Silve et al., 2005). To overcome the challenges involved in identifications of calcium binding sites with weak binding affinity of membrane proteins, Huang et al. developed a computational algorithm to be used for the prediction of potential Ca^2+^ binding pockets based on a statistical analysis of multiple calcium binding proteins (Huang Y. et al., 2007; Kirberger et al., 2008; Huang et al., 2009; Wang et al., 2009, 2010; Zhao et al., 2012). Shown in Figure 3, five potential Ca^2+^-binding sites were predicted in each monomer of the ECD of the homologous model of CaSR. Site 1 (S147, S170, D190, Y218, and E297) is located within the hinge region between the two lobes of the ECD (Kubo et al., 1998; Silve et al., 2005; Huang Y. et al., 2007). It is interesting to note that E297, S147, and S170 were reported to be important for sensing Ca^2+^ and/or amino acids (Bai et al., 1999; Mun et al., 2005; Silve et al., 2005). Recently, a novel Ca^2+^-binding site in the similar hinge region adjacent to the reported L-Glu binding site in mGluR1 was found to synergistically activate the receptor with L-Glu, and enhance the activity of the mGluR1 orthosteric and allosteric ligands (Jiang et al., 2010, 2014). This same phenomenon may also be applicable to other type of cGPCRs.

Since the initial prediction, several complementary approaches have been used to provide important insights into how CaSR functions and the behavior of the receptor at the molecular level. First, functional cooperativity contributed by each Ca^2+^-binding site was determined by site-directed mutagenesis and monitoring intracellular Ca^2+^ oscillations, IP production and ERK_1∕2_ activation in living cells. We reported that the predicted Ca^2+^-binding site 1 within the hinge region of the ECD of CaSR and its interaction with other Ca^2+^-binding sites within the ECD is essential in tuning functional positive homotropic cooperativity of CaSR caused by changes in [Ca^2+^]_o_. Furthermore, molecular dynamic simulations indicated that there is molecular connectivity among the other predicted Ca^2+^-binding sites with site 1 and that the VFT hinge domain plays a central role in functional cooperativity (Zhang et al., 2014a). These results from various functional studies suggest that cooperative binding of Ca^2+^ at multiple predicted Ca^2+^-binding sites of the CaSR likely maximizes its capacity to respond over a narrow physiological range of [Ca^2+^]_o_ independent of amino acids or other agonists (Bai et al., 1998a; Zhang et al., 2001; Bai, 2004; Figure 2)

The direct binding of Ca^2+^ to the other predicted Ca^2+^-binding sites in the ECD of CaSR and related binding cooperativity has been probed by using several methods. Ca^2+^ binding capabilities of predicted Ca^2+^-binding Site 3 (residues E224, E228, E229, E 231 E232), and Site 5, which is formed by contiguous Ca^2+^ binding residues (residues E378, E379, T396, D398, E399), were verified using a grafting approach (Huang Y. et al., 2007). By grafting peptide sequences composed of key predicted Ca^2+^ ligand binding residues separated by flexible linkers into a non-Ca^2+^-binding protein, site specific Ca^2+^ binding capability was determined using a Tb^3+^-sensitized FRET assay. Huang et al. also applied this approach to probe Ca^2+^ binding at three additional Ca^2+^ binding sites and potential binding cooperativity. Three major subdomains of the CaSR containing various numbers of predicted Ca^2+^ binding sites were expressed and purified. Their Ca^2+^-binding capabilities were examined using mutagenesis studies combined with various spectroscopic studies utilizing 8-anilino-1-naphthalenesulfonic acid (ANS) fluorescence, intrinsic tryptophan fluorescence spectra and nuclear magnetic resonance (NMR) (Huang et al., 2009). It was striking to observe that subdomain 1 with three binding sites (including sites 1, 2, and 3) exhibited a large Ca^2+^ dependent conformational change. A binding process with strong and weak Ca^2+^-binding affinities has been clearly unveiled for subdomain 1 by both fluorescence and NMR studies. These studies not only confirmed the existence of multiple Ca^2+^ binding sites, but also revealed that the molecular connectivity among multiple metal binding sites is essential for a highly cooperative functional activity (Figure 2).

The complex glycosylated and high mannose CaSR ECD (residues 20–612) were purified from HEK293S and its mutant cell line (Zhang et al., 2014c). Using various spectroscopic methods, it was shown that both form the ECD bound to Ca^2+^ with a *K*_d_ of 3.0–5.0 mM. The local conformational changes of the proteins induced by their interactions with Ca^2+^ were visualized by NMR with specific ^15^N Phe-labeled forms of the ECD. These studies also suggest that glycosylation does not affect calcium binding properties.

## Identification of amino acid binding site and heterotropic functional activity

The extracellular calcium induced activation of CaSR is potentiated by L-amino acids to maintain the whole body Ca^2+^ homeostasis, making the receptor a multimodal and multimetabolic sensor (Francesconi and Duvoisin, 2004; Breitwieser, 2006; Conigrave et al., 2007a). Under physiological conditions, L-amino acids, especially aromatic amino acids (e.g., L-Phe, L-Trp), as well as short aliphatic and small polar amino acids, potentiate the extracellular calcium triggered CaSR activity by altering the EC_50_ values required for Ca^2+^-evoked intracellular calcium responses and its functional cooperativity. For example, the EC_50_ for Ca^2+^ decreased from 4.2 ± 0.2 to 2.5 ± 0.1 mM in the presence of L-Phe (Conigrave et al., 2000, 2007a; Wang et al., 2006). At the threshold level of extracellular Ca^2+^ concentration, L-amino acids induced slow intracellular Ca^2+^ oscillations with a frequency at around 1–2 peaks/min (Breitwieser, 2006). In aggregate, the levels of amino acids in human serum in the fed state are close to those activating the CaSR *in vitro* (Conigrave et al., 2000, 2007b) and can further enhance functional cooperativity via positive heterotropic cooperativity. The CaSR in cells within the lumen of the gastrointestinal (GI) tract are also activated by L-Phe and other amino acids, which have been long recognized as activators of key digestive processes. Thus, the CaSR enables the GI tract to monitor events relevant to both mineral ion and protein/amino acid metabolism, in addition to its sensing capability in blood and related extracellular fluids (Conigrave et al., 2000; Wang et al., 2006; Broadhead et al., 2011; Liou et al., 2011). Distinctive signaling pathways elicited by amino acids compared with Ca^2+^ have been demonstrated in quite a few studies (Breitwieser, 2006; Broadhead et al., 2011). Ca^2+^ activates CaSR though activation of the heterotrimeric GTP binding protein G_q_, causing the generation of Ins(1,4,5)P3 (IP3), which binds to the IP3 receptor on the ER, releasing stored Ca^2+^. On the other hand, L-Phe in the presence of Ca^2+^ induces coupling to heterotrimeric GTP binding protein G_12∕13_, leading to the activation of RhoA via filamin A.

Some potential L-Phe binding site residues in the CaSR were identified based on the conserved amino acid residues involved in the binding of glutamate to mGluRs (Zhang et al., 2002). Their studies demonstrated that mutating three adjacent Ser (S169, S170, S171) to Ala eliminated L-Phe potentiated receptor activity measured by [Ca^2+^]_i_ mobilization using a fluorescence-based cell population assay. Meanwhile, studies from Mun et al. showed the binding site for amino acids was within the VFT domain of CaSR by utilizing CaSR-mGluR chimeric receptor constructs. The receptor lacking the ECD exhibited impaired response to L-amino acids (Mun et al., 2004). The same group showed that two residues, Thr145 and Ser170, could be crucial for sensing L-amino acids, using mutagenesis and fluorimetry studies (Mun et al., 2005).

Recently, an L-Phe binding site adjacent to the previously predicted calcium-binding site 1 based on computational docking results was reported. The potential L-Phe-binding site is composed of residues Leu51, Thr145, Ser170, Tyr218, and Ser272. The residue Tyr218 is involved in both Ca^2+^ and Phe binding. Extensive mutational studies using both single cell imaging and fluorimetric assays identified the importance of L-Phe-binding pocket for positive heterotropic cooperativity between extracellular Ca^2+^ and L-Phe in eliciting CaSR-mediated Ca^2+^ signaling (Zhang et al., 2014a). The frequency of sinusoidal intracellular Ca^2+^ oscillations in CaSR transfected HEK293 cells not only depend on the extracellular Ca^2+^, but also affected by the binding of allosteric modulators. For example, the binding of CaSR with Ca^2+^ alone produces a intracellular Ca^2+^ oscillations frequency (1.5/min at room temperature) which is different than those produced by Ca^2+^ with L-amino acid (e.g., L-Phe) (2.2/min) (Breitwieser, 2006; Zhang et al., 2014b). This change in frequency is extremely important for the role of CaSR signal transduction in the many different tissue and organ environments in which it is present. For instance, the Ca^2+^ oscillations in parathyroid, kidney, intestine and bone are tightly related to maintenance of Ca^2+^ homeostasis while the Ca^2+^ signals are processed differently in many epithelial cells and the nervous system (Riccardi, 2002; Breitwieser, 2006).

**Figure 4:**
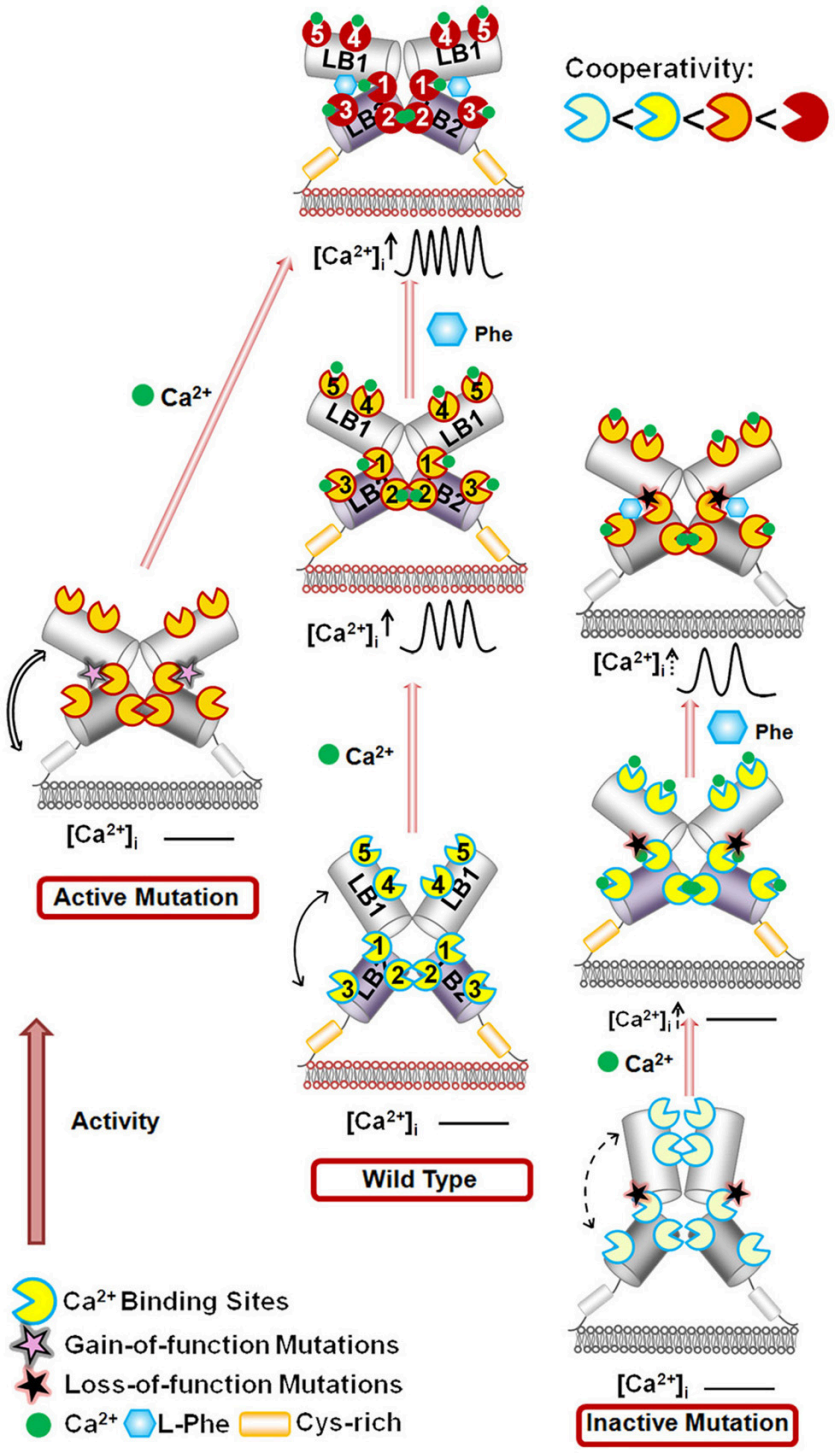
**Schematic representation of the mechanisms underlying the effects of the mutations on the CaSR and the modulation of receptor activity by extracellular Ca^**2+**^ and L-Phe**. Ca^2+^ and L-Phe modulate the activity as well as the cooperativity of CaSR (the color changes of the receptor from ivory to red indicate an increase in functional activity). Elevating [Ca^2+^]_o_, e.g., to 3.0 mM, is proposed to change the basal WT CaSR status into an active form in a positive homotropic cooperative manner and further trigger intracellular Ca^2+^ oscillations. L-Phe binds to the hinge region between lobe 1 and lobe 2, modulating the receptor together with Ca^2+^ in a positive heterotropic cooperative way. This could potentiate conversion of the receptor to a “fully active” form associated with a higher frequency of intracellular Ca^2+^ oscillations and a left-shifted EC_50_. Loss-of-function CaSR mutants (indicated by ivory color) could cause a disruption of the cooperativity among the various Ca^2+^-binding sites as shown by impaired correlation motions (dashed arrows). The impaired receptor function and the cross-talk between Ca^2+^-binding sites can be at least be partially rescued for some mutants by L-Phe (e.g., P221Q). However, if the mutation interferes with the interaction between CaSR and L-Phe, the function of the receptor may not be fully recovered (e.g., L173P). CaSR gain-of-function mutants (left) exhibit enhanced correlated motions (double line arrows) and their activity is not further potentiated by L-Phe, potentially due to a ceiling effect. LB1(2): Lobe 1 or lobe 2 in the VFT domain of CaSR.

The hetero-communication between Ca^2+^ and an amino acid functions as a dual switch that globally enhances functional positive homotropic cooperative activation of CaSR in response to Ca^2+^ signaling by positively impacting multiple Ca^2+^-binding sites within the ECD (Figure 3; Zhang et al., 2014a). A direct interaction between the CaSR ECD and L-Phe was finally reported using saturation transfer difference NMR approaches with a determined binding affinity of ~10 mM in the absence of Ca^2+^ (Zhang et al., 2014c). The study further demonstrated that L-Phe increases the binding affinity of the CaSR ECD for Ca^2+^. Thus, dual binding of calcium and amino acids at the hinge regions of the bilobed VFTD of the CaSR leads to activation of the receptor with highly cooperative responses to the changes in the extracellular concentrations of these agonists (Figure 4).

## Molecular basis of disease related CaSR mutations

More than 200 mutations of the CaSR that lead to the disorders of Ca^2+^ homeostasis have been identified (http://www.casrdb.mcgill.ca/) (Hendy et al., 2009). Mutations of CaSR may inhibit receptor activity, lead to an over activation of the receptor, or markedly impair the receptor's structure. The majority of these disease-associated mutations are missense mutations, with a single amino acid substituted, often from one base pair change. Amino acids insertion, deletion, open reading frame shift, and splice-site mutations have also been reported (Hendy et al., 2009). The diseases associated with inactivate receptors include cases of Familial Hypocalciuric Hypercalcemia (FHH) (Ward et al., 2006) and Neonatal Severe Hyperparathyroidism (NSHPT) (Thakker, 2004) while the disorders associated with the CaSR activating mutations include Autosomal Dominant Hypocalcemia (ADH) (Hendy et al., 2000) and Bartter syndrome type V (Watanabe et al., 2002).

**Figure 5:**
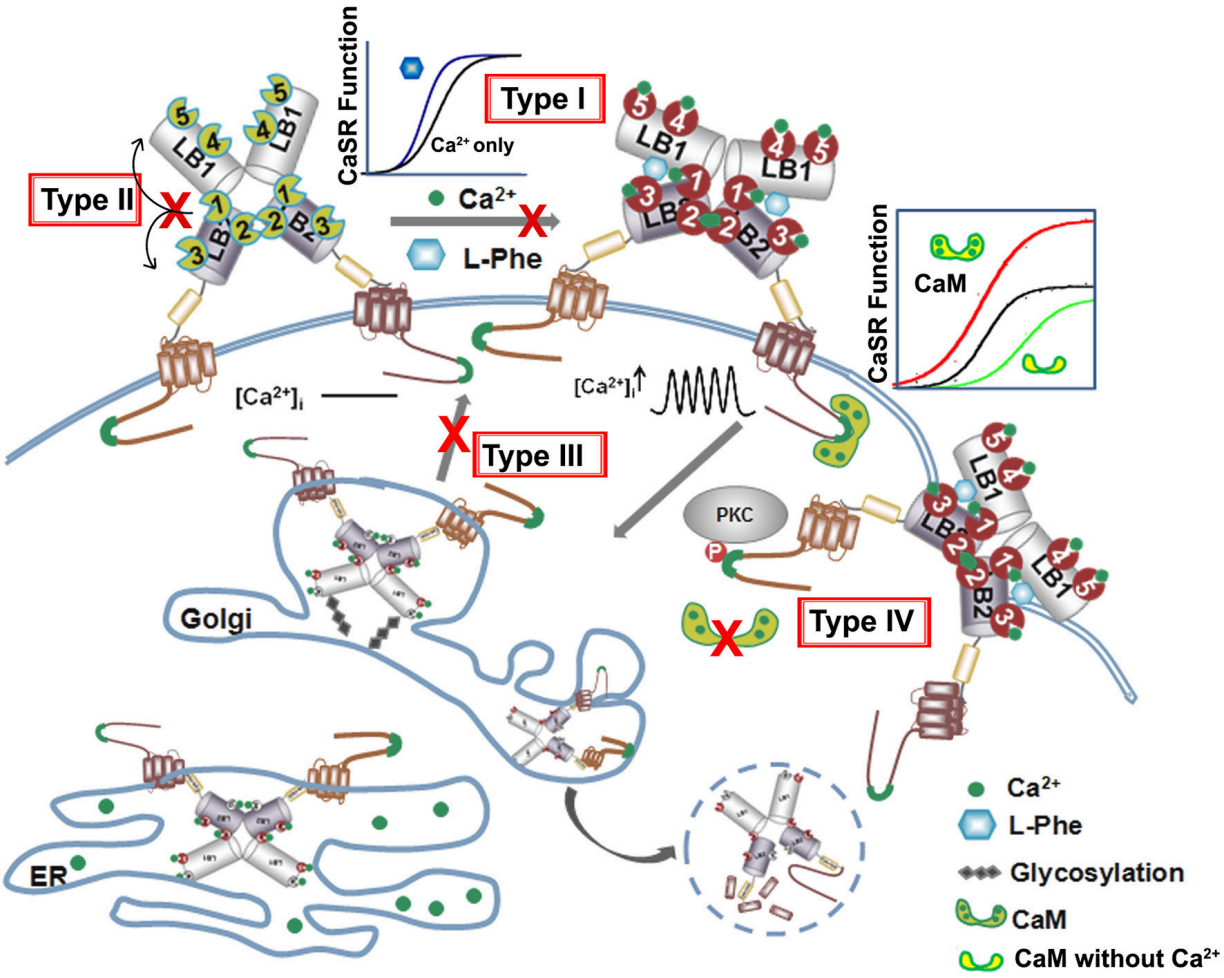
**Summary of four types of disease mutations**. The CaSR trafficking, expression and surface stabilization contribute to its functional cooperativity. Type I disease associated mutations directly alter key calcium and ligand binding capability at or in close proximity of the predicted ligand binding sites in the ECD. Type II mutations alter CaSR function especially EC_50_ or Hill number without altering surface expression or trafficking, but affect the molecular connectivity between different ligand binding sites. Type III mutations disrupt the cooperativity via interfering with the receptor cell surface expression and Type IV mutations largely affect potency of cooperativity by altering trafficking and protein stability via interaction with proteins binding to the CaSR intracellular C-tail.

Below is the summarized four types of disease mutations based on their distinct mechanisms of action. Type I and type II mutations alter the CaSR's function, especially EC_50_ or Hill number without altering surface expression or trafficking. Type III and type IV mutations largely affect degree of cooperativity via altering surface expression and trafficking of the protein to the plasma surface (Figure 5, Table 1).

**Table 1:** **Summarized molecular basis of disease related CaSR mutations**.

**Mutation types**	**Function on CaSR**	**Examples**	**References**
Type I	Mutations directly affecting key Ca^2+^ and ligand binding residues or in close proximity of binding pocket. Leads to a loss-of- or gain-of-function of CaSR.	E297K, E297D, Y218S, Y218C, R227L, E191K, E228Q, E228K, E350V, E354A, D215G, etc.	Pearce et al., 1996; Silve et al., 2005; Deng et al., 2006; Huang Y. et al., 2007; Huang et al., 2009; Hannan et al., 2012; Zhang et al., 2014d
Type II	Mutations disrupting the molecular connectivity embedded into the hinge region of the ECD between other proposed Ca^2+^ sites, without effect on surface expression or intracellular trafficking.	L173F, L173P, P221Q, and P221L.	Felderbauer et al., 2006; Zhang et al., 2014b
Type III	Mutations leading to a decreased or increased potency of cooperativity by altering its cell surface expression. Have lower expression or overexpression on the cell surface of CaSR leads to a change in Ca^2+^-sensing capabilities.	P39A, G143E, L174R, C582Y, R638L, S657Y, P748R, G778D, E297K, C395R, A804D, P55L, S137P, R185Q, D215R, G553R, R795W, V817I, P798T, S657Y, G670E, R680H, M734R, etc.	Bai et al., 1998a; Fan et al., 1998; Zhang et al., 2001; Christopoulos and Kenakin, 2002; Casadó et al., 2007; Huang and Breitwieser, 2007; White et al., 2009; Leach et al., 2013
Type IV	Mutations which effect cooperativity of CaSR by hampering its intracellular trafficking capabilities and protein stability via the intracellular C-terminal tail and its binding partners.	T876ins, F881L, R886W, and R886P for FHH/NSHPT and S895del, del920–970, mutations on partner proteins, etc.	Thakker, 2004; Kemp et al., 2009; Stepanchick et al., 2010; Grant et al., 2011; Arulpragasam et al., 2012; Chakravarti et al., 2012; Davey et al., 2012; Breitwieser, 2013; Nesbit et al., 2013

The first type of mutational effect, type I, is via direct alterations in key calcium and ligand binding capability at or in close proximity to the predicted ligand binding sites in the ECD (Figure 5). Hannan et al. found that more than 50% of their newly identified CaSR mutations in patients with FHH, NSHPT, and ADH are within the ECD of the receptor (Hannan et al., 2012). Analysis using the homology modeled structure of the CaSR further revealed that >50% of these missense substitutions are located within 10 Å of one or more proposed calcium-binding sites, indicating that the bilobed VFT domain plays a pivotal role in interacting with Ca^2+^ and regulating the function of CaSR (Hannan et al., 2012). Intriguingly, more than half of the mutations near Ca^2+^-binding sites are situated close to Site 1, suggesting the importance of the VFT domain cleft. The molecular connectivity between Site 1 and rest of the calcium-binding sites can possibly facilitate the positive cooperativity of the CaSR (Zhang et al., 2014d).

Through Ca^2+^ binding site modeling and mutational studies, several key residues for the predicted Ca^2+^ binding sites in the hinge region of the VFT domain have been identified (Deng et al., 2006; Huang Y. et al., 2007; Huang et al., 2009). Amongst them, more than half of the ECD mutations are near the proposed Ca^2+^ binding sites, and a few of them are found directly in the potential binding site residues. One ADH mutation (E297D) and several FHH mutations (Y218S, Y218C, and E297K) are within Ca^2+^ binding site 1. The FHH mutation D215G is located in Ca^2+^ binding site 2 while two ADH mutations E228Q and E228K are in Site 3. The Ca^2+^ binding Site 4 embraces residues involved in a carcinoma-associated mutation E350V and an ADH mutation E354A. Mutations on these key binding residues leads to a decrease of the EC_50_ of Ca^2+^ in ADH in order to produce PTH, and an increase of EC_50_ in FHH or NSHPT. This can also lead to an alteration of binding cooperativity between the other Ca^2+^ binding sites, allowing them to bind more or less effectively as indicated by changes in the Hill number. Several studies have investigated the effect of FHH Ca^2+^ binding site 1 mutations, such as Y218S (Pearce et al., 1996) and E297K (Bai et al., 1999), both of which led to a loss-of-function with an increase in EC_50_ for Ca^2+^ in terms of intracellular Ca^2+^ mobilization. Additionally, E297K also decreases inositol-1-phosphate (IP1) accumulation, but does not change CaSR cell expression levels as compared to the wild-type (WT) (Bai et al., 1999). ADH mutation E297D, as well as FHH mutation E297K, were analyzed by Silve et al. for IP1 accumulation and expression levels (Silve et al., 2005). Again, they saw no change in cell expression compared to the WT, but they did see an activation shift for E297D, as the EC_50_ for Ca^2+^ induced IP stimulation changed from the WT EC_50_ of 4.30 ± 0.20 to 2.70 ± 0.30 mM, and a loss-of-function shift in EC_50_ for E297K (Silve et al., 2005).

Additionally, mutations in residues neighboring the binding site also show the same trend in an altered EC_50_ and cooperativity but with no apparent effect on cell surface expression or trafficking, such as the FHH/NSHPT mutations S171N, R227Q, R227L, S296N, F351V, W352X, C395R, G397R, the ADH mutations E191K, E241K, Q245R, and cancer-associated mutations, S169F and S171G. Mutations on these binding site residues or their neighboring residues would have major effects on either direct binding or conformational direction of the binding site residues, respectively. Pearce et al. looked at two such mutants R227L (NSHPT) and E191K (ADH) (Pearce et al., 1996). R227L increased EC_50_ for extracellular Ca^2+^ induced intracellular Ca^2+^ response to 9.3 mM compared to the WT 4.0 mM, while the E191K mutant decreased EC_50_ for [Ca^2+^]_o_ from the WT 3.7 to 2.8 mM. Western blot analysis showed that compared to WT there was no increase or loss in expression (Pearce et al., 1996). Thus, concluding that binding site residues, and their immediate bordering residues, significantly alter the charge or conformation of the binding pocket therefore altering functionality into a gain-of or loss-of-function mutation with a major effect on protein expression, translation, and/or trafficking.

The second type of disease mutations, type II, disrupts molecular connectivity embedded into the hinge region of the ECD (Figures 3, 5). Mutations at the particular region of the N-terminal VFT domain produce either receptor inactivation (L173P, P221Q) or activation (L173F, P221L) related to hypercalcemic or hypocalcemic disorders. We have shown that both L173P and P221Q markedly impair the functional positive cooperativity of the CaSR as reflected by Ca^2+^–induced intracellular Ca^2+^ oscillations, IP_1_ accumulation and extracellular signal-regulated kinases (ERK_1∕2_) activity. In contrast, L173F and P221L show enhanced responsiveness of these three functional readouts to [Ca^2+^]_o_ change. Further analysis of the dynamics of the VFT domain mutants using computational simulation studies supports disruption in the correlated motions in the loss-of-function CaSR mutants, while these motions are enhanced in the gain-of-function mutants. WT CaSR was modulated by L-Phe in a heterotropic positively cooperative way, achieving an EC_50_ for Ca^2+^ induced intracellular responses similar to those of the two activating mutations. The response of the inactivating P221Q mutant to Ca^2+^ was partially rescued by L-Phe, illustrating the capacity of the L-Phe binding site to enhance the positive homotropic cooperativity of CaSR. L-Phe had no effect on the other inactivating mutant. Moreover, our results carried out both *in silico* and within intact cells indicate that residue Leu173, which is adjacent to residues that are part of the L-Phe-binding pocket, exhibited impaired heterotropic cooperativity in the presence of L-Phe. Thus, P221 and L173 are important for the positive homo—and heterotropic cooperative regulation elicited by agonist binding.

The third type of mutations, type III, directly affect CaSR protein surface expression level which, in turn, alters potency or cooperativity. Apparent functional cooperativity and the EC_50_ for extracellular Ca^2+^ elicited intracellular response of the CaSR are also determined by the receptor expression level at the plasma membrane (Christopoulos and Kenakin, 2002; Casadó et al., 2007; Huang Y. et al., 2007). Like many other membrane proteins, CaSR's surface expression level is determined by the rate of internalization and folding in the endoplasmic reticulum (ER) and Golgi, and membrane insertion. The CaSR protein is synthesized in the ER and is trafficked intracellularly in the ER and Golgi complex as it undergoes translations in the ER and post-translational modifications before shuttling to the cell surface. Interruptions in this trafficking from translation to cell surface expression, such as those from disease mutations, can lead to loss-of or gain-of-function CaSR on the cell surface, CaSR that is retained in the ER or Golgi, or degraded altogether. Breitwieser's group investigated how these disease mutations affect trafficking as a whole. They identified several classes of mutations, spanning the whole CaSR length, which they categorized based on their ability to traffic to the cell surface and to have their function rescued by the positive allosteric modulator NPS R-568. Class Ib mutations (Q27R, P39A, R66C, G143E, L174R, G549R, C582Y, R638L, S657Y, G670E, P748R, G778D, L849P) interrupt trafficking so substantially that the CaSR is not able to leave the ER, while Class Ia mutations (E297K, C395R, and A804D) similarly remain in the Golgi complex. All Class I mutations fail to traffic to the cell surface. Class II mutations still allows CaSR to reach the cell surface and their function either can be enhanced by NPS R-568 (Class IIa: P55L, R62M, S137P, R185Q, D215R, R227Q, G553R, L650P, R680C, V689M, R795W, V817I) or not (Class IIb: L159P and P798T) (Huang and Breitwieser, 2007; White et al., 2009). Consistent with these observations, Leach et al. reported that FHH mutations S657Y, G670R, G670E, R680H, R680C, M734R, G778D, and V817I significantly decrease cell surface expression compared to the WT; while ADH mutations F821L, V836L, and A843E increase cell surface expression, although the ADH mutation F832S did lead to a 45% decrease in surface expression (Leach et al., 2013).

The 14 cysteines found in the ECD and the cys-rich domain were also found to affect CaSR function and cell surface expression (Bai et al., 1998a; Fan et al., 1998; Zhang et al., 2001). Of these 14 cysteines, 9 have disease mutations associated with them producing ADH, FHH, and various carcinomas. C129S and C131S, the cysteines thought to participate in the homodimerization of CaSR through disulfide bond formation, only led to a decrease of expression to 88 and 97%, respectively, compared to WT CaSR. Whereas, C60S led to a decrease in expression by half, and all other Cys mutations linked to disease led to a decrease to lower than 28% or undetectable levels (Fan et al., 1998). It is clear that the disruption of disulfide formation as well as trafficking have profound effects on functional cooperativity via alterations in both the Hill number and the concentration of active form on the plasma surface.

The fourth mutational effect, type IV, alters CaSR interactions with trafficking and stabilization partners leading to a change in surface expression that further influences CaSR signaling as well as functional cooperativity. Breitwieser et al. have concluded that agonist-driven insertional signaling (ADIS) contributed to the hallmarks of CaSR signaling, including the high degree of cooperativity and the lack of functional desensitization. Additionally, they have shown that the life cycle of the CaSR controls the cellular abundance of CaSR, suggesting an intimate link between trafficking and signaling (Breitwieser, 2012, 2013). As shown in Figure 5 and Table 2, a large number of proteins have been reported to interact at the C-terminal tail, the protein interaction sensitive zone, of the CaSR (Hebert and Brown, 1995; Chakravarti et al., 2012; Brown, 2013). Interactions of these proteins may be important for both CaSR signaling and functional cooperativity. Among these proteins, E3 ubiquitin ligase, dorfin is linked to ubiquitination and degradation of CaSR via binding to C-terminal amino acids 880–968 (Huang et al., 2006) and/or the C-terminal amino acids 920–970 (Zhuang et al., 2012). Zhang et al. investigated the effect of lysosomal mediated degradation of CaSR C-terminus and found that loss of the region 920–970 led to the highest cell expression level and decrease in degradation (Zhuang et al., 2012). Several insertion and deletion mutations in these regions of the C-terminus of CaSR lead to ADH, resulting in reduced degradation and, therefore, over-expression at the cell surface. Additionally, we have reported that the surface expression of CaSR is largely influenced by interaction of calmodulin (CaM) at residues 871–898 at the C-terminal tail of CaSR. Interestingly, multiple disease mutations, such as T876ins, F881L, R886W, and R886P for FHH/NSHPT and S895del for ADH reside at this CaM binding region (Huang et al., 2010). CaM has been reported to bind both the immature and mature forms of CaSR, which suggests its role in modulating anterograde trafficking (Bai et al., 1998b). We have shown that deletion of the CaM binding region significantly abolishes/reduces functional cooperativity of calcium oscillations. Therefore, CaM binding stabilizes the receptor on the cell membrane and thus increases potency of its functional activity (Huang et al., 2010). It is worth pointing out that this region also is involved in phosphorylation and biased signaling (Davey et al., 2012; Leach et al., 2012). Moreover, protein 14-3-3 was reported to interact with the arginine-rich domain of CaSR (^890^RRxxxxRKR^898^), which may lead to the retention of CaSR in the ER (Stepanchick et al., 2010; Grant et al., 2011; Arulpragasam et al., 2012). CaSR associated disorders can also be caused by different mechanisms other than direct mutations on the receptor itself. Recent studies by Nesbit et al. and Rogers et al. revealed that disorders with FHH or ADH phenotype can be associated with mutations on partner proteins associated with CaSR-mediated signaling, for instance the loss-of-function mutations of the sigma subunit of adaptor protein-2 (AP2) and mutations on G-protein subunit-α11 (GNA11), (Nesbit et al., 2013; Rogers et al., 2014). Moreover, antibodies against the CaSR can also regulate the function of the receptor (Thakker, 2004; Kemp et al., 2009, 2010).

**Table 2:** **The calcium-sensing receptor interacting proteins**.

**Protein**	**Assay used**	**Function**	**CaSR domain**	**References**
AMSH	Y2H, GST	Trafficking/de-Ub enzyme	C-terminal	Herrera-Vigenor et al., 2006
β-Arrestin	Functional	Trafficking/signaling	Unknown	Lorenz et al., 2007
Caveolin	Co-IP	Structural/scaffolding/ trafficking/signaling	Intracellular loop 1 and 3	Kifor et al., 1998, 2003; Sun and Murphy, 2010; Breitwieser, 2013
E3 Ub ligase	Y2H, IP, functional	Trafficking	C-terminal	Huang et al., 2006
Filamin	Y2H, Co-IP, GST, functional	Structural/scaffolding/trafficking	C-terminal	Awata et al., 2001; Hjälm et al., 2001; Pi et al., 2002
GRK-2	Functional	Signaling	ICD	Lorenz et al., 2007
GRK-4	Functional	Signaling	ICD	Lorenz et al., 2007
Kir4.1	Y2H, Co-IP, functional	K channel	C-terminal	Huang C. et al., 2007
Kir4.2	Y2H, Co-IP, functional	K channel	C-terminal	Huang C. et al., 2007
PI-4-Kinase	Co-IP,	Signaling	Unknown	Huang et al., 2002
PKC	Functional	Signaling	C-terminal	Lorenz et al., 2007
RAMP1	Co-IP, functional	Structural/trafficking	ECD and 7TM	Bouschet et al., 2005
RAMP3	Co-IP, functional	Structural/trafficking	ECD and 7TM	Bouschet et al., 2005
RGS proteins	Functional	Signaling	Unknown	Huang et al., 2002, 2004
Rho	Co-IP	Signaling	Unknown	Huang et al., 2002
14-3-3	Yeast two-hybrid screen, CaSR tail pull-down studies, Co-IP	Trafficking, expression, signaling	Proximal membrane region, CaSR C-terminal tail	Grant et al., 2011; Arulpragasam et al., 2012
CaM	GST, Co-IP, functional	Signaling/stabilize	C-terminal	Huang et al., 2010
P24A	Co-IP	Stabilize/ trafficking	C-terminal	Stepanchick and Breitwieser, 2010
Sar1	Functional	Trafficking	ECD	Zhuang et al., 2010
Rab1, Rab7, Rab 11a	Functional, RNAi	Trafficking/signaling	Possible ECD	Zerial and McBride, 2001; Grosshans et al., 2006
Dorfin	Co-IP, functional	Trafficking	Intracellular loop, C-terminal	Huang et al., 2006
Integrins	Co-IP, proteomic analysis, co-localization, functional	Cell migration and adhesion in cancer cells, trafficking, signaling	Unknown	Tharmalingam et al., 2011

## Additional mechanisms to alter the functional activity of CaSR

There are several possible reasons why a single CaSR could play a major role in various organisms, organs and tissue types at different physiological environments. First, besides Ca^2+^, other divalent cations such as Mg^2+^, Ba^2+^, Mn^2+^, Ni^2+^, Sr^2+^ (Thomsen et al., 2012), and trivalent cations La^3+^ and Gd^3+^ can also interact at similar reported calcium binding sites, and/or in their local vicinity, via altered electrostatic interactions of the protein. In general, the higher the positive charge density, the higher the potency as CaSR agonists. The CaSR has a relatively low affinity for Mg^2+^ with an EC_50_ at about 10 mM as measured for ligand induced current in oocytes, whereas there has been a reported EC_50_ of 3 mM for Ca^2+^ in the same assay (Brown et al., 1993). Mg^2+^ also has only half the maximal capacity for the production of inositol phosphate and arachidonic acid when stimulated by Ca^2+^ in CHO cells transiently expressing rat CaSR (Ruat et al., 1996). Consistently, CaSR transiently expressed in HEK293 cells has EC_50_s for intracellular calcium responses of 15–20 and 3–5 mM for Mg^2+^ and Ca^2+^ under physiological conditions, respectively (Bai et al., 1996; Nearing et al., 2002). CaSR ligands are also important for therapeutics, for example, Sr^2+^ (as a ranelate salt) has been shown to be effective for the treatment of osteoporosis (Kendler et al., 2009). CaSR has also been seen to respond to binding ligands (e.g., Ca^2+^, Mg^2+^, L-Phe, etc.) on a pH dependent manner where slightly lower extracellular pH of 7.2 inhibits the binding of the ligand to CaSR, and a higher pH of 7.6 favors the binding process (Campion et al., 2015).

Second, various classes of agonists, antagonists and drugs can also regulate the activation of CaSR via the ECD. The established homotropic cooperativity and heterotropic co-activation model of CaSR can also explain the regulatory action on CaSR. Three basic peptides including poly-arginine, protamine, and poly-lysine exhibited a dose-dependent inhibition of dopamine-stimulated cAMP accumulation in dispersed bovine parathyroid cells with EC_50_ values at micromolar or sub-micromolar concentrations (Brown et al., 1991). Studies from Quinn et al. demonstrated alteration of several cellular parameters including [Ca^2+^]_i_ change, IP production, and the activity of a nonselective cation channel via polyamines. The potency of polyamines was directly proportional to number of positive charges with the order of spermine > spermidine > > putrescine (Quinn et al., 1997). Glutathione and its γ-glutamyl peptides also allosterically modulate the CaSR at a site that appears to be similar to the L-amino acids binding site but with >100-fold higher apparent affinity (Conigrave et al., 2000; Wang et al., 2006; Broadhead et al., 2011). The EC_50_ values for GSH and the oxidized form glutathione disulfide (GSSG), to induce intracellular Ca^2+^ mobilization, were in the range of sub-micromolar (0.08 and 0.33 μM, respectively) (Wang et al., 2006). In 2006, using [Ca^2+^]_i_ mobilization, PTH secretion, as well as intracellular cAMP inhibition, Broadhead et al. showed γ-glutamyl-tripeptides (γ-Glu-Cys-Gly, S-methylglutathione and S-propylglutathione, and dipeptides γ-Glu-Ala and γ-Glu-Cys) to be positive allosteric modulators of CaSR. They also found that the double mutant T145A/S170T had an impaired response to these peptides, indicating a potential peptide binding site at the extracellular domain of CaSR (Broadhead et al., 2011). Recently, a peptide drug AMG 416 has been shown to act as an agonist of the CaSR and is undergoing clinical trials (Walter et al., 2013). Interestingly, the action of AMG 416 is also modeled to be at the hinge region of the Phe-Site1 calcium binding site (Alexander et al., 2015). Mutations on Cys482 at the hinge region lead to impaired peptide activity in both pig and human CaSR (Alexander et al., 2015).

Third, calcium sensitivity could be related with the difference in CaSR sequence observed in various species. In mammals, CaSRs were detected in multiple organ systems besides parathyroid glands (e.g., kidneys, colon, skin, parathyroid, stomach, vascular, bone, etc.), participating in various functions, such as Ca^2+^ and fluid reabsorption, acid secretion, osteoblast and keratinocyte differentiation, etc. (Tfelt-Hansen and Brown, 2005; Alfadda et al., 2014). The CaSR sequences among major clades exhibit few differences both at the ECD and ICD. The length of the ICD varies greatly among the clades (Herberger and Loretz, 2013). Intriguingly, the sequence of the putative calcium binding sites, especially the Site 1 within the ECD thought to be the main Ca^2+^-binding domain, is relatively conserved among vertebrates (Huang et al., 2009). On the other hand, residues in the other predicted Ca^2+^-binding sites differ substantially, which may be an effective way to adjust CaSR sensitivity to a specific physiological environment, i.e., with varying Ca^2+^, Mg^2+^, or pH, and also for evolution. For example, CaSR is present in different organs of fish, suggesting it might play a crucial role in sensing the changes in the salinity in surrounding water with calcium concentrations changes from 10 mM in sea water to 0.07–2.0 mM in freshwater. The presence of many alternatively spliced forms of the human CaSR could imply an impact of these altered sequences on the CaSR's Ca^2+^ sensitivity (Oda et al., 1998, 2000).

Fourth, Ca^2+^ signaling could be modulated through the formation of CaSR hetero-dimers with other GPCRs or higher order oligomers with non GPCR chaperones in tissues other than parathyroid. Gama et al. showed co-localization of the CaSR and mGluR1α in hippocampal and cerebellar neurons by immunoprecipitation of CaSR from bovine brain. The CaSR became sensitive to glutamate triggering internalization and exhibited altered trafficking via reported hetero-dimerization with GABA_B_ receptors (Gama et al., 2001). Thus, it was speculated that the CaSR would respond to Ca^2+^ concentration within the synaptic cleft at various levels of synaptic activity. Recent work by Kim et al. revealed that CaSR forms a heteromeric complex with the inhibitory type B γ-aminobutyric acid receptor 1 (GABA_B_R1) in hippocampal neurons (Kim et al., 2011). This study demonstrates a novel receptor interaction, which contributes to ischemic neuron death through CaSR upregulation and GABA_B_R1 downregulation, and feasibility of neuroprotection by concurrently targeting these two receptors. It is also a new way to tailor functional cooperativity and specificity.

## Conclusion and perspective

Recently, the crystal structure of extracellular domain has been first solved by our group. We reported the first CaSR crystal structure with multiple Mg^2+^ binding sites (Zhang et al., 2016). Consistent to our modeled structure reported earlier, the determined X-ray structure of the ECD shares the same fold of the mGluRs with the VFT motif. Unexpectedly, we also identified a tryptophan derivative L-1,2,3,4-tetrahydronorharman-3-carboxylic acid (TNCA) at the hinge region between the two subdomains where orthosteric ligand binding is thought to occur. We further demonstrated that TNCA binds to CaSR at unusually high affinity and potentiates CaSR activity with Ca^2+^ and Mg^2+^ (Zhang et al., 2016). Geng and colleagues subsequently reported X-ray structures of CaSR ECD in both apo form and the Ca^2+^ binding form. Similar as proposed in earlier studies, CaSR active form contained multiple binding sites for Ca^2+^. Additional Ca^2+^ bound sites and ${\text{PO}}_{4}^{3-}$ binding sites were reported. They also showed that L-Trp bound to the orthosteric agonist-binding site in CaSR active form (Geng et al., 2016). These advances and various pioneer studies since the discovery of CaSR in understanding the cooperative extracellular Ca^2+^ signaling mediated through CaSR have uncovered important structural and functional characteristics about this receptor. Family cGPCRs all sense extracellular calcium with differing degrees and also exhibit sensitivity to either amino acids, neurotransmitters, or related ligands. The discovered co-activation by calcium and amino acids of CaSR is likely to have a broad impact for the regulation of cGPCRs. However, many questions about CaSR are still unanswered. Though accelerated elucidation of cGPCR structures have been accomplished, the unavailability of the full CaSR structure and largely “invisible loop residues” in the determined structure of cGPCRs limit the next step forward on understanding of molecular mechanism of CaSR. Thus, high resolution structures of CaSR with various forms of agonists and antagonists are essential to gain a better understanding of the underlying mechanism in CaSR regulated physiological functions as well as pathological activities. Equal importance should be directed at studies designed to probe the functional cooperativity of CaSR as it plays a pivotal role in controlling the receptor response within a narrow fluctuation of metal concentration. Family cGPCRs are highly relevant for drug design and have tremendous potential as therapeutic targets because cGPCRs play vital roles in neurotransmitter release and Ca^2+^ homeostasis. The aforementioned results, along with further determination of the structure of CaSR, will provide great insights into the molecular basis of the structure and function of CaSR and shed new light on other cGPCRs in health and disease states. Thus, further uncovering of the structural and functional mysteries of CaSR could aid the development of novel receptor-based therapeutics to use in the treatment of many different diseases.

## Author contributions

JY, CZ, CM, JZ, and RG contributed to the manuscript's conception and wrote the manuscript. KH performed computational analysis. EB made searches and helped with the manuscript.

## Funding

This work was supported by National Institutes of Health Grants GM081749 and EB007268 (to JY), and funds from the Georgia Research Alliance.

### Conflict of interest statement

The authors declare that the research was conducted in the absence of any commercial or financial relationships that could be construed as a potential conflict of interest.
